# Prime Time Light Exposures Do Not Seem to Improve Maximal Physical Performance in Male Elite Athletes, but Enhance End-Spurt Performance

**DOI:** 10.3389/fphys.2017.00264

**Published:** 2017-05-01

**Authors:** Raphael Knaier, Juliane Schäfer, Anja Rossmeissl, Christopher Klenk, Henner Hanssen, Christoph Höchsmann, Christian Cajochen, Arno Schmidt-Trucksäss

**Affiliations:** ^1^Division Sports and Exercise Medicine, Department of Sport, Exercise and Health, University of BaselBasel, Switzerland; ^2^Basel Institute for Clinical Epidemiology and Biostatistics, University Hospital Basel and University of BaselBasel, Switzerland; ^3^Centre for Chronobiology, Psychiatric Hospital of the University of BaselBasel, Switzerland; ^4^Transfaculty Research Platform Molecular and Cognitive Neurosciences, University of BaselBasel, Switzerland

**Keywords:** circadian rhythm, bright light, blue light, melatonin, chronotype

## Abstract

Many sports competitions take place during television prime time, a time of the day when many athletes have already exceeded their time of peak performance. We assessed the effect of different light exposure modalities on physical performance and melatonin levels in athletes during prime time. Seventy-two young, male elite athletes with a median (interquartile range) age of 23 (21; 29) years and maximum oxygen uptake (VO2max) of 63 (58; 66) ml/kg/min were randomly assigned to three different light exposure groups: bright light (BRIGHT), blue monochromatic light (BLUE), and control light (CONTROL). Each light exposure lasted 60 min and was scheduled to start 17 h after each individual's midpoint of sleep (median time: 9:17 pm). Immediately after light exposure, a 12-min time trial was performed on a bicycle ergometer. The test supervisor and participants were blinded to the light condition each participant was exposed to. The median received light intensities and peak wavelengths (photopic lx/nm) measured at eye level were 1319/545 in BRIGHT, 203/469 in BLUE, and 115/545 in CONTROL. In a multivariate analysis adjusted for individual VO2max, total work performed in 12 min did not significantly differ between the three groups. The amount of exposure to non-image forming light was positively associated with the performance gain during the time trial, defined as the ratio of the work performed in the first and last minute of the time trial, and with stronger melatonin suppression. Specifically, a tenfold increase in the exposure to melanopic light was associated with a performance gain of 8.0% (95% confidence interval: 2.6, 13.3; *P* = 0.004) and a melatonin decrease of −0.9 pg/ml (95% confidence interval: −1.5, −0.3; *P* = 0.006). Exposure to bright or blue light did not significantly improve maximum cycling performance in a 12-min all-out time trial. However, it is noteworthy that the estimated difference of 4.1 kJ between BRIGHT and CONTROL might represent an important performance advantage justifying further studies. In conclusion, we report novel evidence that evening light exposure, which strongly impacts the human circadian timing system, enables elite athletes to better maintain performance across a 12-min cycling time trial.

## Introduction

Many athletes reach their peak endurance performance between the afternoon and the early evening (Reilly and Waterhouse, 2009) depending on their chronotype (Facer-Childs and Brandstaetter, 2015). However, in professional sports, competitions very often take place in the late evening (08:00 p.m.–12:00 a.m.) to comply with prime time on television as just recently shown during the Summer Olympic Games 2016 with many finals taking place between 10:00 p.m. and 00:25 a.m. During this time window, circadian related increases in melatonin levels and sleep propensity are expected, which have detrimental effects on cognitive (Schmidt et al., 2007) and physical performance (Facer-Childs and Brandstaetter, 2015). Exposure to artificial light, however, can shift circadian melatonin rhythms (Gronfier et al., 2004; Wirz-Justice et al., 2004; Revell et al., 2006), acutely lower melatonin levels, increase alertness (Cajochen, 2007), and improve mood (Hoffmann et al., 2008a). The extent of these effects depends on intensity (Zeitzer et al., 2000; Hoffmann et al., 2008a), wavelength (Cajochen et al., 2005; Vandewalle et al., 2007; Hoffmann et al., 2008a,b; Smith et al., 2009; Chellappa et al., 2011; Rüger et al., 2013), individual light-dark history (Hébert et al., 2002), duration (Chang et al., 2012), and time of day of light exposure (Cajochen, 2007). Blue light with a wavelength of 460–480 nm activates the non-image forming system with particularly strong effects on alertness, melatonin, and thermoregulation (Cajochen et al., 2005).

Depending on the type and duration of light exposure as well as the population tested, light exposure studies showed contradictory effects on physical performance in bicycle ergometer time trials. First, there was no statistically significant difference in maximum power output during a 45-s time trial following a 90-min light exposure to 5,000 lx compared to 50 lx in young males (Ohkuwa et al., 2001). Second, exposing young males during a 20-min time trial to either 2,788 lx or 6,434 lx compared to 1,411 lx showed no differences in maximum power output (O'Brien and O'Connor, 2000). Performance-enhancing effects may have been masked by testing long-distance runners in a very short time trial on a bicycle ergometer (Ohkuwa et al., 2001) and by the high-intensity light exposure (1,411 lx) used in the control condition (O'Brien and O'Connor, 2000; i.e., ceiling effect). Third, light exposure to 2,500 lx for 30 min in the evening compared to 0 lx significantly increased power output in a 10-km time trial taking place the next morning (Thompson et al., 2015). None of these studies took time-of-day effects or individual circadian rhythms into account.

In a recent study (Kantermann et al., 2012) a 120-min light exposure to 4,420 lx, starting ~14:45 h after the individual midpoint of sleep, significantly increased total work during a 40-min time trial compared to 230 lx. Further, we were able to demonstrate a dose-response relationship between light exposure and physical performance (Knaier et al., 2016), such that longer durations and higher intensities lead to higher power output in a 40-min time trial. However, compared to participants in the control light group, the bright light group only performed significantly more work during the first 24 min of the time trial (Knaier et al., 2016) indicating relatively higher effects for shorter time trials. Thus, for this study time trial duration was set at 12 min, also because this duration is known by most Swiss and German athletes from the Cooper-Test (12 min duration) and represents more competitions (e.g., 5,000 m) than a 40-min time trial. Further, the cardiorespiratory fitness test was expected to last approximately the same duration and served therefore as familiarization.

However, the studies conducted so far still have a number of limitations: assessors were not blinded for type of light exposure (O'Brien and O'Connor, 2000; Ohkuwa et al., 2001; Kantermann et al., 2012; Thompson et al., 2015), melatonin as an accepted marker for circadian phase was not measured (O'Brien and O'Connor, 2000; Ohkuwa et al., 2001; Kantermann et al., 2012), sample size was small (O'Brien and O'Connor, 2000; Ohkuwa et al., 2001; Thompson et al., 2015), examined participants had a relatively low peak aerobic performance (Thompson et al., 2015), and potential confounding factors such as sleep quality and sleep duration or drugs (e.g., caffeine consumption) were not (O'Brien and O'Connor, 2000; Ohkuwa et al., 2001) or only partially monitored (Kantermann et al., 2012; Thompson et al., 2015). Further, the influence of blue light on physical performance has not yet been investigated.

Thus, the aim of this study was to assess the effect of both bright and blue light exposure on physical performance in elite athletes under well controlled conditions to take into account numerous potential confounders. Our primary hypothesis was that exposure to bright (BRIGHT) or blue light (BLUE) prior to a time trial in the late evening would increase work performed in elite endurance athletes compared to a control condition (CONTROL). Secondary aims were to assess the effect of the different light conditions and intensities on melatonin levels, sleepiness, and mood.

## Materials and methods

### Study design

**Figure 1:**
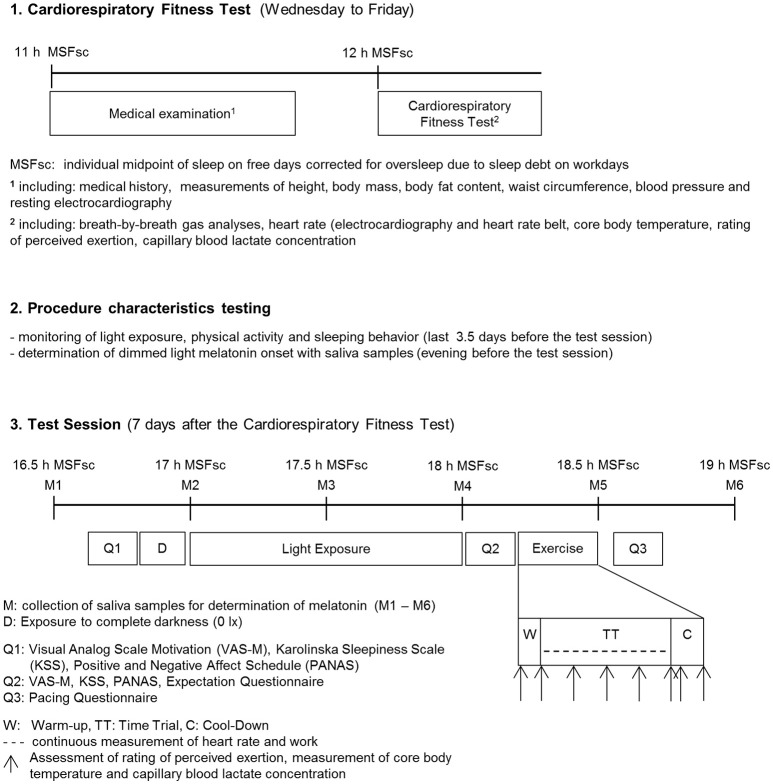
**Experimental procedure**.

This 3-arm parallel group randomized controlled trial was conducted between April 2014 and April 2015 in the laboratories of the Department of Sport, Exercise and Health of the University of Basel, Switzerland (ClinicalTrials.gov Identifier: NCT02203539). The study was approved by the local ethics committee (Ethikkommission Nordwest- und Zentralschweiz 2014-056). Written informed consent was obtained from all study participants before the start of study. We used permuted block randomization with randomly varying block sizes of 3, 6, and 9 to allocate participants at random and in equal numbers to one of the three groups. The randomization list was generated in advance using the online tool available at http://www.randomization.com (accessed April 29, 2014) and transmitted using sequentially numbered, opaque, sealed envelopes. A graphical abstract of the study design is provided (Figure 1).

### Participants

Physically healthy men between 18 and 35 years of age, with no shift-work in the last 3 months and no travels across time zones in the last 4 weeks before the study, were recruited for a baseline cardiorespiratory fitness test to determine aerobic exercise capacity by measuring maximum oxygen uptake (VO_2_max). Only elite endurance athletes with VO_2_max ≥ 55 ml/kg/min were invited to complete the test session 6–8 days later. Athletes were randomized into the three groups bright light (BRIGHT), blue light (BLUE), and control light (CONTROL). All tests were conducted in the same laboratory by the same test supervisor (R.K.) to guarantee comparability between groups. Further, the tests were only carried out from Wednesday to Friday to ensure that participants had a regular sleeping routine for a minimum of three nights before the test session. To avoid a wide variability in the participants' light-dark history, no tests were carried out for 30 days before and after the 21st of June (longest day of the year). No participants were tested in the week after the change to daylight saving time.

### Participant characteristics testing

At baseline, a clinical examination was performed, including medical history and a physical examination consisting of measurements of height, body mass, body fat content, waist circumference, blood pressure, and resting electrocardiography. Motivation was measured by a 10 cm visual analog scale and sleep quality was assessed by the Pittsburgh Sleep Quality Index (Buysse et al., 1989). The Munich Chronotype Questionnaire (Roenneberg et al., 2004) was used to determine individuals' midpoint of sleep on free days corrected for oversleep due to sleep debt on workdays (MSFsc). Then a cardiorespiratory fitness test until exhaustion was conducted on a bicycle ergometer (Sport Excalibur, Lode Medical Technology, Groningen, The Netherlands) starting 12 h after the individual MSFsc. After a 5 min warm-up phase at 50 W, workload increased linearly with 25 W/min until exhaustion, followed by a 5 min cool-down phase at 50 W. The protocol is expected to achieve VO_2_max according to previous findings (Midgley et al., 2008). Pedaling cadence was chosen by participants but was required to be over 60 revolutions per minute. Participants were allowed to cycle with their own pedals and shoes. Breath by breath gas analyses (MetaMax 3B, Cortex Biophysik GmbH, Leipzig, Germany) and heart rate (12-channel electrocardiography, Custo med GmbH, Ottobrunn, Germany and additionally a Polar T-34 heart rate belt, Polar Electro Europe AG, Zug, Switzerland) were measured continuously, tympanic temperature and rating of perceived exertion according to the 6–20 Borg scale (Borg, 1982) were assessed at rest, after warm-up and every 3 min until exhaustion. Blood pressure and capillary blood lactate concentration (analyzed by SuperGL Ambulance, Hitado Diagnostic Systems, Moehnesee, Germany) were measured at rest, immediately after exhaustion and during the cool-down phase at 3 min after exhaustion. Exhaustion was only accepted if all of the following four criteria were fulfilled: [1] Respiratory exchange ratio ≥1.1; [2] blood lactate concentration >8 mmol/l (Steinacker et al., 2002); [3] rating of perceived exertion ≥19; and [4] maximum heart rate >95 % of predicted maximum heart rate [210—age (years)].

### Procedure characteristics testing

Participants were advised to refrain from alcohol, nicotine, caffeine, chocolate, bananas, sport, and visits to the solarium during the last 2 days before the test session. Further, they were encouraged to keep a constant sleeping routine (i.e., bedtime ± 1 h) during the 3 days prior to the test session. To monitor compliance regarding sleeping routine and restraint from sport, participants wore two wGT3X+ ActiGraphs (Pensacola, United States, measuring rate of 60 Hz) 24 h per day during the last 3.5 days before the test session. One device was worn on the non-dominant hand above the clothes measuring light exposure and one was worn on the waist measuring physical activity. During this time period participants also kept a diary recording sunlight exposure and physical activity to double-check data from the ActiGraph, sleeping habits during the last 3 days and nutrition during the last 2 days before the test session to monitor if melatonin-affecting substances such as bananas, alcohol etc. were consumed. On the evening before the time trial, participants collected five saliva samples every 60 min, starting 4 h before individual bedtime under dimmed light (i.e., <50 lx) to determine the participants' dim-light melatonin onset with the hockey stick method (Danilenko et al., 2014). Saliva samples were frozen at −24°C until analysis for melatonin (pg/ml) via radioimmunoassay (Bühlmann Laboratory, Schönenbuch, Switzerland).

### Test session

Six to eight days after the baseline test eligible participants were randomly assigned to one of the three different light exposure groups: BRIGHT, BLUE, and CONTROL. All participants were exposed to darkness (0 lx) for 10 min before light exposure to dark adapt the participants' pupils. Light exposure was scheduled to start 17 h after the individual MSFsc and lasted 60 min. The three light conditions were BRIGHT with two Philips Energylight HF3319 devices with ~4,400 lx in total and peak wavelength of 545 nm (range: 400–720 nm; Philips, Eindhoven, The Netherlands), BLUE with two Philips goLITE BLU devices with ~230 lx in total and peak wavelength of 469 nm (range: 440–520 nm; Philips, Eindhoven, The Netherlands) and CONTROL with two Philips Energylight HF3319 devices with ~230 lx in total and peak wavelength of 545 nm (range: 400–720 nm; Philips, Eindhoven, The Netherlands). The lamps were placed on a table at a distance of 60 cm from the participants' eyes.

As different sitting postures and the direction of gaze have influence on the amount of light reaching the eye, individually perceived light intensity was recorded with a sensor (LUXBlick 2.0, Technische Universität Ilmenau, Ilmenau, Germany) with a sampling rate of 1 Hz that was attached to glasses, which participants wore during the light exposure. Participants were not given any information about the alternative study groups and the light sources were only referred to as “Light 1,” “Light 2,” and “Light 3.” Every light setting was arranged in a different room in order to blind the test supervisor (RK) to the light condition each participant was going to be exposed to. The door of each room was labeled with “Light 1,” “Light 2,” or “Light 3.” Participants were allocated at random to one of the three light conditions. Participants were asked to go into the room specified in the envelope. Additionally, two timers were handed out, one indicating when participants had to take the next saliva sample and the other one to show the end of the light exposure. After the end of the light exposure participants immediately returned to the laboratory. From the recorded illuminance levels measured in photometric lux via the “LUXBlick” glasses worn by the participants, the amount of melanopic lux reaching the eye was calculated for each participant (Lucas et al., 2014) to quantify the influence of the non-image forming photoreceptor system on performance. Salivary melatonin was measured 60 min prior to and until 2 h after the start of light exposures at 30 min intervals.

After the light exposure, a questionnaire about the expected effect of the light exposure on the work performed during the time trial was filled out. Sleepiness was assessed with the Karolinska Sleepiness Scale (Kaida et al., 2006), motivation with the visual analog scale, and mood with the Positive And Negative Affect Schedule (Crawford and Henry, 2004) directly before and immediately after light exposure. Following light exposure, athletes performed a 12-min time trial on a bicycle ergometer. The bicycle ergometer test started 18:15 h after MSFsc with a 2-min warm-up phase at 50 W followed by a 12-min time trial and a 3-min cool-down phase at 75 W. At rest, at the end of warm-up, every 3 min during the time trial, and in the first and third minute of the cool-down phase RPE was assessed. Heart rate was measured continuously. Participants were advised to “bike as far (to generate as much work) as possible” during the 12 min. Workload (Power) and cadence were set at 80% of VO_2_max as assessed during the baseline cardiorespiratory fitness test. To ensure that participants could pedal with the favored cadence, workload increased quadratically (factor α) with increasing pedaling cadence according to the formula: Power = α (Cadence)^2^. The time trial was followed by a questionnaire assessing the participants' pacing strategy during the time trial, on a 10 cm visual analog scale ranging from 0 = “very bad pacing” to 10 = “very good pacing.”

### Statistical analysis

The primary outcome of this study was work performed during the 12-min time trial on a bicycle ergometer; secondary outcomes were sleepiness, motivation, and mood after the light exposure. We used analysis of covariance with adjustment for maximum exercise capacity (VO_2_max) to compare the work performed during the time trial between elite athletes in BRIGHT, BLUE, and CONTROL groups (Vickers and Altman, 2001). Similarly, we used analysis of covariance to compare the secondary outcomes after the light exposure between athletes in BRIGHT, BLUE, and CONTROL adjusted for the corresponding values before the light exposure. Normality was assessed using normal quantile-quantile plots of the residuals and variance homogeneity was assessed using Tukey-Anscombe Plots. For each analysis, we report the estimated differences (with 95% confidence intervals) in outcome between the three groups. We carried out two additional analyses. First, we used a mixed model for repeated measures to estimate whether the light exposure conferred a differential effect on physical performance at different time points during the time trial. In this analysis, we used a first-order autoregressive structure for the covariances among the 12 min of the time trial for each participant, while different participants were still assumed to be independent. Second, we used linear regression to estimate the effect of the amount of exposure to non-image forming (i.e., melanopic) light on the “performance gain” during the time trial, defined as the ratio of the performance during the first and last minute of the time trial. In this analyses eight participants in group BLUE and one participant in group CONTROL were excluded, because they received considerably lower exposure to melanopic light than planned in the protocol. For our analyses and graphics, we used IBM SPSS Statistics for Windows, version 22 (IBM Corp., Armonk, N.Y., USA) and R version 3.3.1 (R Foundation for Statistical Computing, Vienna, Austria), respectively.

### Sample size

For sample size calculation, we assumed that the work performed in BRIGHT, BLUE, and CONTROL was 228 kJ, 232 kJ, and 220 kJ, respectively, and that the standard deviation was 10 kJ (Díaz et al., 2012; Giles et al., 2012; Kantermann et al., 2012). By adjusting for maximum exercise capacity (VO_2_max), we could expect to further reduce error variability and therefore conservatively assumed a correlation of 0.3 between the maximum exercise capacity and work performed during the time trial. With a 2-sided significance level of 0.05, the sample size needed to attain a targeted power of 80% for showing superiority of BRIGHT over CONTROL was 23 participants per group. A total sample size of 3^*^23 = 69 participants gave a power of 97.5% for the overall comparison between the three groups, a power of 98.8% for the comparison between BLUE and CONTROL and a power of 28.8% for the comparison between BRIGHT and BLUE. We anticipated a drop-out rate of 15% and that 10% of athletes assessed for eligibility would not fulfill the inclusion criteria and therefore aimed at recruiting a total of 90 athletes.

## Results

### Participant flow and characteristics

Eighty-seven participants were assessed for eligibility. Of the 74 participants that met the inclusion criteria and were equally randomized to BRIGHT, BLUE, and CONTROL, two had to be excluded after randomization because it was uncertain if they had reached the inclusion criterion of maximum oxygen uptake (VO_2_max) equal or greater than 55 ml/kg/min due to an invalid VO_2_max measurement. Further, three participants had to be excluded from the analysis of the primary outcome (total work performed during time trial) due to invalid measurement of VO_2_max, no exhaustion during the cardiorespiratory fitness test and premature termination of the time trial, respectively. In the cardiorespiratory fitness test, all included participants showed a VO_2_max in the top 10% and the median VO_2_max was in the top 1% of the participants' sex and age group (American College of Sports Medicine, 2010). Characteristics of the included participants were balanced between the three groups (Table 1).

**Table 1:** **Participant characteristics**.

**Characteristic**	**BRIGHT (*n* = 24)**	**BLUE (*n* = 24)**	**CONTROL (*n* = 24)**
Age (years)	23 (22; 30)	23 (21; 26)	24 (23; 30)
Height (cm)	181 (178; 183)	179 (172; 184)	180 (176; 186)
Body mass (kg)	74 (70; 78)	75 (66; 80)	72 (69; 76)
BMI (kg/m^2^)	22 (21; 24)	23 (22; 24)	22 (21; 24)
Body fat (%)	10 (8; 13)	11 (10; 14)	10 (8; 12)
Waist circumference (cm)	79 (75; 82)	78 (74; 81)	78 (75; 82)
Heart rate at rest (bpm)	63 (58; 73)	54 (50; 66)	59 (54; 67)
Pmax (W)	418 (401; 439)	393 (366; 454)	398 (375; 420)
VO_2_max^1^ (ml/kg/min)	64 (61; 66)	60 (57; 66)	62 (59; 65)
VO_2_max^1^ (l/min)	4.83 (4.30; 5.04)	4.55 (4.24; 5.14)	4.48 (4.34; 4.68)
**BLOOD PRESSURE (mmHg)**			
Systolic	130 (125; 135)	130 (122; 134)	125 (120; 130)
Diastolic	80 (75; 89)	80 (76; 85)	78 (75; 85)
**CHRONOTYPE**			
MSFsc (hh:mm)	3:54 (3:24; 5:00)	4:30 (3:54; 5:18)	4:12 (3:42; 4:36)
DLMO^2^ (hh:mm)	20:30 (19:48; 21:36)	21:18 (20:24; 22:00)	20:54 (20:30; 21:42)
**SMOKING (%)**			
Never smoker	92	92	92
Former smoker	8	8	8
Current smoker	0	0	0
**MAIN SPORT (%)**			
Bike / Triathlon	58	42	50
Other endurance	9	25	13
Game	25	33	21
Other	8	0	16

*BRIGHT, bright light; BLUE, blue light; CONTROL, control light; BMI, body mass index; Pmax, maximum power output during cardiorespiratory fitness test; VO_2_max, maximum oxygen uptake; MSFsc, mid-sleep on free days corrected for “oversleep” due to the sleep debt accumulated over the workweek; DLMO, dim light melatonin onset*.

1 *Available in 23 (96%) and 23 (96%) participants in BRIGHT and BLUE, respectively*.

2 *Available in 21 (88%), 19 (79%) and 18 (75%) participants in BRIGHT, BLUE and CONTROL, respectively. Data are median (interquartile range) if not stated otherwise*.

### Procedure characteristics and time trial performance

Participants were well circadian-entrained as indexed by a normal phase angle between the timing of melatonin onset and habitual bedtime and showed normal sleep quality as well as regular sleep patterns during the last 3 days before the time trial. Motivation was rated rather high (Table 2).

**Table 2:** **Procedure characteristics**.

**Characteristic**	**BRIGHT (*n* = 24)**	**BLUE (*n* = 24)**	**CONTROL (*n* = 24)**
**CARDIORESPIRATORY FITNESS TEST**			
Start (hh:mm after MSFsc)	11:48 (11:30; 12:30)	12:00 (11:30; 12:42)	11:36 (11:00; 11:54)
VAS-M (cm)	9.4 (8.5; 10)	9.5 (8.6; 10)	9.4 (8.5; 10)
PSQI	3 (2.8; 4)	3 (1.8; 4)	3 (1; 3.2)
**EXHAUSTION CRITERIA**			
HRmax^1^ (bpm)	193 (186; 200)	192 (185; 196)	192 (188; 200)
RER^2^	1.16 (1.14; 1.2)	1.19 (1.16; 1.21)	1.21 (1.17; 1.22)
RPE^3^	20 (20; 20)	20 (20; 20)	20 (20; 20)
Blood lactate^3^ (mmol/l)	13.2 (12.1; 15)	15.7 (13.7; 16.6)	14.9 (12.9; 15.7)
**LIGHT EXPOSURE**			
Photopic (lx)	1326 (960; 1591)	202 (29; 598)	115 (78; 208)
Melanopic (lx)	1159 (839; 1390)	2173 (319; 6414)	100 (68; 182)
**TIME TRIAL**			
Start (hh:mm after MSFsc)	18:16 (18:15; 18:18)	18:16 (18:14; 18:18)	18:16 (18:15; 18:18)
PSQI	2.5 (2; 4.2)	3 (2; 4.2)	2.5 (1; 4)
**SLEEP (hh:mm)**			
Mid-sleep TT-3	3:30 (2:42; 4:42)	3:30 (3:00; 4:06)	3:18 (2:54; 4:00)
Mid-sleep TT-2	3:12 (2:30; 4:06)	3:42 (3:00; 4:12)	3:24 (2:54; 4:00)
Mid-sleep TT-1	3:24 (3:00; 4:06)	3:42 (3:12; 4:24)	3:30 (3:00; 4:18)
Sleep duration TT-3	7:48 (7:24; 8:36)	7:30 (6:48; 8:48)	7:42 (6:42; 8:18)
Sleep duration TT-2	7:30 (6:36; 8:00)	7:24 (6:54; 8:48)	7:30 (7:00; 8:00)
Sleep duration TT-1	7:36 (6:42; 8:18)	7:36 (7:00; 8:24)	7:42 (7:06; 8:24)

*BRIGHT, bright light; BLUE, blue light; CONTROL, control light; MSFsc, mid-sleep on free days corrected for “oversleep” due to the sleep debt accumulated over the workweek; VAS-M, Visual Analog Scale Motivation; PSQI, Pittsburgh Sleep Quality Index; HRmax, maximum heart rate; RER, respiratory exchange ratio; RPE, rate of perceived exertion; TT-3, three nights before the time trial; TT-2, two nights before the time trial; TT-1, one night before the time trial*.

1 *Available in 22 (92%) participants in BLUE*.

2 *Available in 23 (96%) and 23 (96%) participants in BRIGHT and BLUE, respectively*.

3 *Available in 23 (96%) participants in BLUE*.

*Data are median (interquartile range)*.

Work performed during the time trial was highest in BRIGHT followed by BLUE and CONTROL, with an average (standard deviation) of 229 (23), 218 (34), and 216 (25) kJ, respectively (Table 3). In the multivariate analysis adjusted for the pre-specified potential confounder VO_2_max, the difference in work performed during the time trial was 4.1 kJ (95% confidence interval [CI] −4.5, 12.7; *P* = 0.346) for participants in BRIGHT and −1.2 kJ (95% CI −9.8, 7.5; *P* = 0.787) for participants in BLUE, both relative to participants in CONTROL (Table 3).

**Table 3:** **Analysis of covariance to determine the effects of light exposure on physical performance**.

	**BRIGHT (*n* = 24)**	**BLUE (*n* = 23)**	**CONTROL (*n* = 24)**	**BRIGHT vs. CONTROL**	**BLUE vs. CONTROL**	**BRIGHT vs. BLUE**
**Characteristic**	**Time trial [mean (*SD*)]**	**Time trial [mean (*SD*)]**	**Time trial [mean (*SD*)]**	**Adjusted difference^2^^,^^3^ (95% CI)**	**Adjusted difference^2^^,^^3^ (95% CI)**	**Adjusted difference^2^^,^^3^ (95% CI)**
Work^1^ (kJ)	229 (23)	218 (34)	216 (25)	4.1 (−4.5; 12.7)	−1.2 (−9.8; 7.5)	5.3 (−3.4; 14)

*BRIGHT, bright light; BLUE, blue light; CONTROL, control light; SD, standard deviation; CI confidence interval*.

1 *Available in 23 (96%) participants in BLUE—one participant terminated the time trial prematurely*.

2 *Work (kJ) adjusted for maximum oxygen uptake (VO_2_max, l/min)*.

3 *Analysis based on 69 (96%) participants—one participant in BLUE terminated the time trial prematurely and two participants in BRIGHT and BLUE had invalid VO_2_max measurements in the cardiorespiratory fitness test [definitely above 55 ml/kg/min (inclusion criterion)]*.

In an additional repeated measures analysis adjusted for the individual VO_2_max, we added the factor “time on trial” and determined whether there was a statistically significant “group” (BRIGHT, BLUE, CONTROL) x “time on trial” interaction effect on performance (*P* = 0.235).

**Figure 2:**
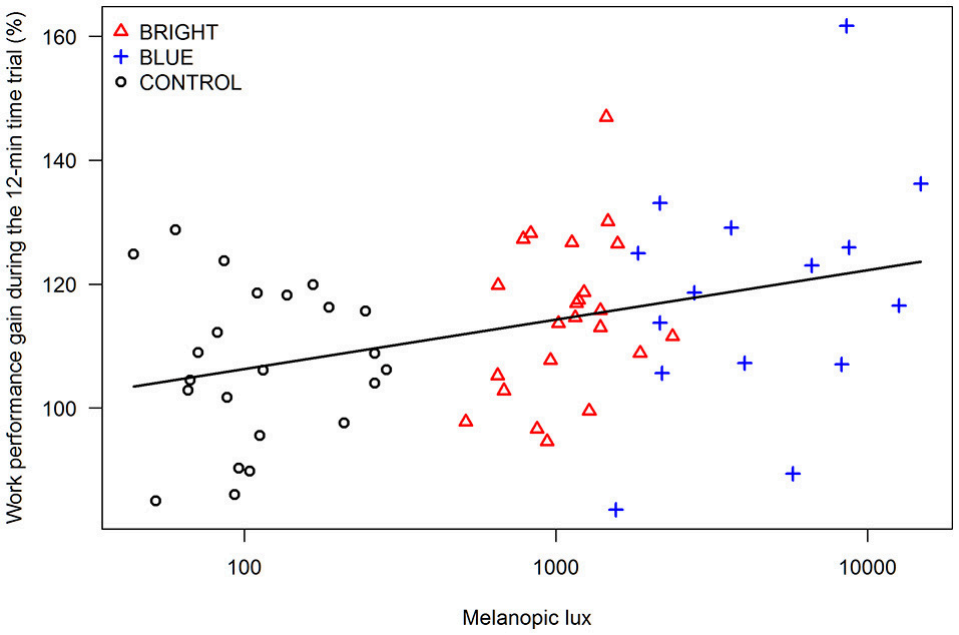
**Correlation between the amount of exposure to non-image forming light (i.e., melanopic lx) and the “performance gain” (in %) during the time trial, defined as the ratio of the performance in the first and last minute of the time trial (BRIGHT = bright light, BLUE = blue light, and CONTROL = control light)**.

The median melanopic light exposure (calculated for each participant individually from the photometric light intensity measured on eye level) was 1,153 lx (interquartile range [IQR] 829, 1,390), 2,173 lx (IQR 335, 7,041) and 100 lx (IQR 68, 182) in BRIGHT, BLUE, and CONTROL, respectively. The amount of exposure to non-image forming light (i.e., melanopic light) was positively associated with the “performance gain” during the time trial, defined as the ratio of the performance in the first and last minute of the time trial. A tenfold increase in the exposure to melanopic light was associated with an increase in performance gain of 8.0% (95% CI 2.6, 13.3, *P* = 0.004; Figure 2).

### Effect of light exposure on melatonin

**Figure 3:**
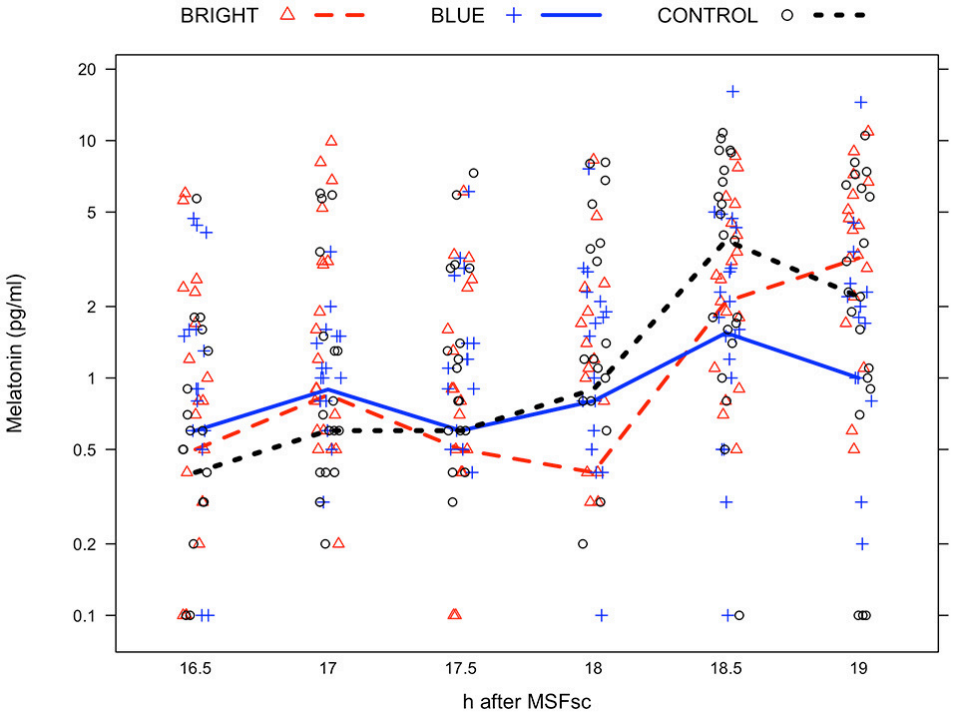
**Median saliva melatonin concentration (in pg/ml) in each group (BRIGHT = bright light, BLUE = blue light, and CONTROL = control light) at 16.5 h (i.e., 30 min before the light exposure), 17 h (i.e., immediately before the light exposure), 17.5 h (i.e., 30 min after the start of the light exposure), 18 h (i.e., immediately after the light exposure), 18.5 h (i.e., immediately after the time trial), and 19.0 h (i.e., 30 minutes after the time trial) after MSFsc (mid-sleep on free days corrected for “oversleep” due to the sleep debt accumulated over the workweek)**. The maximum number of missing melatonin samples per time point and light exposure group was 3/24 (12%), with a total of 16/360 (4%) samples missing.

Immediately after the light exposure melatonin suppression was strongest in BRIGHT followed by BLUE and CONTROL with a median of 0.4, 0.8, and 0.9 pg/ml, respectively (Figure 3). When adjusting for melatonin levels before the light exposure, the difference in melatonin levels after the light exposure, but before the time trial, was −1.1 pg/ml (95%CI −2.2, 0.0) for participants in BRIGHT and −0.5 pg/ml (95%CI −1.6, 0.6) for participants in BLUE, both relative to participants in CONTROL. Similarly, when adjusting for melatonin levels before the light exposure, the amount of exposure to melanopic light was negatively associated with melatonin levels after the light exposure. A tenfold increase in the exposure to melanopic light was associated with a decrease in melatonin by −0.9 pg/ml (95% CI −1.5, −0.3; *P* = 0.006).

### Effect of light exposure on sleepiness, motivation, and mood

The difference in sleepiness between participants in BRIGHT and BLUE was −1.2 points on the Karolinska Sleepiness Scale (95% CI −1.9; −0.4), indicating higher alertness effects by bright rather than blue light at the beginning of the time trial. There was no statistically significant effect of light exposure on motivation or the positive or negative domain of the positive and negative affect schedule (Table 4).

**Table 4:** **Analysis of covariance to determine the effects of light exposure on sleepiness, motivation and mood**.

	**BRIGHT (*n* = 24)**		**BLUE (*n* = 24)**		**CONTROL (*n* = 24)**		**BRIGHT vs. CONTROL**	**BLUE vs. CONTROL**	**BRIGHT vs BLUE**
**Characteristic**	**Before [mean (*SD*)]**	**After [mean (*SD*)]**	**Before [mean (*SD*)]**	**After [mean (*SD*)]**	**Before [mean (*SD*)]**	**After [mean (*SD*)]**	**Adjusted difference^1^ (95% CI)**	**Adjusted difference^1^ (95% CI)**	**Adjusted difference^1^ (95% CI)**
KSS	3.2	3.1	2.9	4	3	3.5	−0.6	0.6	−1.2
	(1)	(1.2)	(1.2)	(1.7)	(0.8)	(1.4)	(-1.3; 0.2)	(−0.2; 1.4)	(−1.9; −0.4)
VAS-M	8.9	8.7	8.7	8.6	9.3	9.2	−0.2	−0.1	−0.1
(cm)	(1)	(1.4)	(1.7)	(1.6)	(0.8)	(0.7)	(-0.6; 0.2)	(−0.5; 0.3)	(−0.5; 0.3)
PANAS	3.6	3.6	3.7	3.4	3.7	3.5	0.1	−0.1	0.3
–(pos)	(0.4)	(0.6)	(0.5)	(0.7)	(0.4)	(0.6)	(-0.1; 0.4)	(−0.4; 0.1)	(0; 0.5)
PANAS	1.5	1.4	1.4	1.2	1.5	1.3	0.1	0	0.1
–(neg)	(0.5)	(0.4)	(0.4)	(0.2)	(0.5)	(0.3)	(0; 0.2)	(−0.2; 0.1)	(0; 0.2)

BRIGHT, bright light; BLUE, blue light; CONTROL, control light; KSS, Karolinska Sleepiness Scale; VAS-M, Visual Analog Scale Motivation; PANAS, Positive and Negative Affect Schedule; SD, standard deviation; CI confidence interval.

1 *Scale after the light exposure adjusted for the corresponding value before the light exposure*.

### Adverse effects and expectations about light exposure

Only minor adverse effects due to the light exposure, such as slight headaches or getting tired, were reported (BRIGHT: *n* = 4; BLUE: *n* = 2; CONTROL: *n* = 4). Expectations about the impact of the light exposure on performance during the time trial differed between the three groups, with 38% of participants in BLUE expecting a decrease in performance and 13% in BRIGHT and 21% in CONTROL. Participants in all three groups generally rated their pacing strategy as very good.

## Discussion

### Effect of light exposure on performance, melatonin, and sleepiness

In this randomized controlled trial, exposure to bright light before a 12-min time trial on a bicycle ergometer induced a stronger though statistically non-significant performance enhancement as well as greater reductions in sleepiness and melatonin levels in elite athletes than exposure to blue or control light. However, BRIGHT showed the highest performance in the second half of the time trial compared to BLUE and CONTROL. Competitions at this duration e.g., 5,000 m running or 4 km cycling do markedly benefit from the ability to conduct a strong second half, and exercise science has demonstrated this as the preferred pacing strategy (Tucker et al., 2006; Corbett, 2009).

In our study all three groups showed a favorable pattern of the pacing strategy in the 12-min time trial, but group BLUE had the strongest gain in performance compared to CONTROL and BRIGHT, respectively. This finding is further supported by a statistically significant correlation between the amount of exposure to melanopic light and the performance gain during the time trial (Figure 2). A tenfold increase in exposure to melanopic light was associated with 8.0% increase in performance gain across the 12 min time trial. The amount of evening exposure to melanopic light correlates well with the degree of melatonin suppression and light's alerting action (Cajochen et al., 2005). This reduction in melatonin may have caused a delay of the opening of the sleep gate, which usually takes place around the onset of melatonin secretion in the evening (i.e., 08:00 p.m.–00:00 a.m., depending on chronotype) and enables athletes to prevent the time-of-day-related drop in performance. Thus, we have first evidence that late-evening melanopic light exposure allows athletes to perform an enhanced end-spurt compared to moderate light conditions, most likely by a strong activation of the human circadian timing system. Although pacing was positively influenced in BLUE, no superiority over CONTROL in the total work performed during the time trial was observed. This may result from the smaller light emitting device used in BLUE compared to the ones used in CONTROL and BRIGHT going along with a higher likelihood to look past the light source leading to higher variations in the received amount of light within this group.

Yet even though our findings are “small” the reported difference of 4.1 kJ between control and bright light are relevant in competitive sport: a cyclist exposed to control light with a VO_2_max of 4.5 l/min, 180 cm height, 73 kg body mass would be expected to perform 214 kJ in a 12-min time trial, equivalent to a mean velocity of 41.1 km/h and a total distance covered of 8,220 m. If exposed to bright light the expected work performed would be 218 kJ (+1.9%), resulting in a total distance of 8,280 m, thus 60 m more in 12:00 min. The distance of 8,220 m would be covered in 11:54.8 min and thus 5.2 s faster which represents a substantial improvement in a competitive sport environment.

In BRIGHT, participants showed a higher reduction of melatonin than in BLUE and CONTROL. This reduction was also reflected in a lower subjective sleepiness ratings indicating higher alerting effects of bright light compared to blue light exposure. In contrast, many previous studies reported a superiority of blue light over bright light on various physiological parameters (e.g., alertness, melatonin reduction, mood), even at lower doses than those used in this study (Zeitzer et al., 2000; Cajochen et al., 2005; Smith et al., 2009; Chellappa et al., 2011; Rüger et al., 2013). In a previous study from our laboratory (Knaier et al., 2016), participants were exposed to the same intensity and duration of bright and control light as in the current study, but at an earlier time point in respect to the participants' MSFsc (14:30 h), thus 3:45 h earlier. Those participants showed a small positive effect on physical performance during the first 12 min of the time trial [plus 2 kJ (95% CI −4, 8)]. Compared to those results, the effect of bright light on work performed in the present study is higher [plus 4 kJ (95% CI −5, 13)], which is remarkable because the level of cardiorespiratory fitness of the current study population was considerably higher (median VO_2_max = 63 ml/kg/min vs. 56 ml/kg/min). This higher level of fitness is important, since effects of performance enhancement often get smaller with higher athletic level of the examined participant (Wenger and Bell, 1986). A possible reason for the higher effect through bright light despite a higher level of cardiorespiratory fitness may be the longer time interval between the MSFsc and the start of the time trial in the current study (18:15 vs. 14:30 h) meaning that athletes were closer to bed rest at the time of testing.

Interestingly in CONTROL adverse events were reported as often as in BRIGHT. While getting tired (*n* = 2 in CONTROL) could be expected due to the low light intensity the reported slight headaches (*n* = 2 in CONTROL) have not been reported in previous studies and are not explainable. However, since all reported adverse effects of light exposure were only minor, this indicates that bright as well as blue light exposure is safe to use in this population. After the light exposure more participants in group BLUE (38%) expected the light to decrease performance than in CONTROL (21%), although there were no significant differences in subjective sleepiness between the groups. This may be explained by the smaller light device used in BLUE compared to CONTROL or that the participants were irritated by the color of the light.

### Strengths and limitations

To our knowledge this is the largest trial on light exposure and aerobic exercise performance in elite athletes. Light exposure was monitored by objective means for the 3 days prior to the time trial. Further, actual light exposure in the laboratory was measured at the eye level, which has never been done before. Sleep quality and physical activity as potential confounders were also controlled in parallel with light exposure prior to the time trial. MSFsc was assessed with an established questionnaire, melatonin was measured to determine the dimmed-light melatonin onset and to assess the melatonin suppressing effects of light to objectively control for the chronotype of the study participants. Additionally, mood, sleepiness, and expectations associated with the light exposure were assessed with standardized questionnaires. However, since 36 out of 72 participants were not cyclists, they could have benefited from a familiarization trial. Further, we are aware that a realistic competitive situation cannot be reproduced in a laboratory study since e.g., nervousness and anxious arousal may override the effects of light exposure on performance. Thus, our results should be interpreted with care before the background of the transfer to competitive sports.

## Conclusions

Our study provides novel evidence that exposure to non-image forming light can provide elite athletes with a potentially meaningful enhancement of performance in short duration competitions taking place late at night. Our data indicate reduced sleepiness and suppressed melatonin levels as underlying mechanisms. High doses of melanopic light may activate the circadian system and thus compensate for an unfavorable chronotype. The findings from this trial may have a strong impact on the usage of bright light with respect to time of competition, but also clearly show that more data are needed to obtain more precise estimates of the performance-enhancing effect of bright light exposure.

## Author contributions

Conceptualization: AS and CC: Methodology: RK, JS, AS, and CC; Formal Analysis: RK, JS, and CC; Investigation: RK, AR, CK, HH, and AS; Writing original draft: RK, JS, AS, CC, and CH; Writing–Review and Editing: AR, CK, and HH; Visualization: JS, RK, CH, and CC; Supervision: AS

## Funding

This study was supported by the Bundesamt für Sport, Eidgenössisches Departement für Verteidigung, Bevölkerungsschutz und Sport, Switzerland (Grant number: BASPO VM-100121).

### Conflict of interest statement

The authors declare that the research was conducted in the absence of any commercial or financial relationships that could be construed as a potential conflict of interest. JS has been an employee of F. Hoffmann-La Roche Ltd since December 1, 2016. The present study was conducted before JS joined F. Hoffmann-La Roche Ltd and has no connection to her employment by the company.

## Abbreviations

BRIGHT: bright light
BLUE: blue light
CONTROL: control light
VO2max: maximum oxygen uptake
MSFsc: midpoint of sleep on free days corrected for oversleep due to sleep debt on workdays.

## References

[B1] American College of Sports Medicine (2010). ACSM's Guidelines for Exercise Testing and Prescription. 8th Edn. Baltimore, MD: Lippincott Williams & Wilkins.

[B2] Borg G. (1982). Ratings of perceived exertion and heart rates during short-term cycle exercise and their use in a new cycling strength test. Int. J. Sports Med. 3, 153–158. 10.1055/s-2008-1026080 7129724

[B3] Buysse D. J. Reynolds C. F. III. Monk T. H. Berman S. R. Kupfer D. J. (1989). The Pittsburgh sleep quality index: a new instrument for psychiatric practice and research. Psychiatry Res. 28, 193–213. 10.1016/0165-1781(89)90047-4 2748771

[B4] Cajochen C. (2007). Alerting effects of light. Sleep Med. Rev. 11, 453–464. 10.1016/j.smrv.2007.07.009 17936041

[B5] Cajochen C. Münch M. Kobialka S. Kräuchi K. Steiner R. Oelhafen P.. (2005). High sensitivity of human melatonin, alertness, thermoregulation, and heart rate to short wavelength light. J. Clin. Endocrinol. Metab. 90, 1311–1316. 10.1210/jc.2004-0957 15585546

[B6] Chang A. Santhi N. St Hilaire M. Gronfier C. Bradstreet D. Duffy J.. (2012). Human responses to bright light of different durations. J. Physiol. 590, 3103–3112. 10.1113/jphysiol.2011.226555 22526883 PMC3406393

[B7] Chellappa S. L. Steiner R. Blattner P. Oelhafen P. Götz T. Cajochen C. (2011). Non-Visual effects of light on melatonin, alertness and cognitive performance: can blue-enriched light keep us alert? PLoS ONE 6:e16429. 10.1371/journal.pone.0016429 21298068 PMC3027693

[B8] Corbett J. (2009). An analysis of the pacing strategies adopted by elite athletes during track cycling. Int. J. Sports Physiol. Perform. 4, 195–205. 10.1123/ijspp.4.2.195 19567923

[B9] Crawford J. R. Henry J. D. (2004). The Positive and Negative Affect Schedule (PANAS): construct validity, measurement properties and normative data in a large non-clinical sample. Br. J. Clin. Psychol. 43, 245–265. 10.1348/0144665031752934 15333231

[B10] Danilenko K. V. Verevkin E. G. Antyufeev V. S. Wirz-Justice A. Cajochen C. (2014). The hockey-stick method to estimate evening dim light melatonin onset (DLMO) in humans. Chronobiol. Int. 31, 349–355. 10.3109/07420528.2013.855226 24224578

[B11] Díaz V. Peinado A. B. Vleck V. E. Alvarez-Sánchez M. Benito P. J. Alves F. B.. (2012). Longitudinal changes in response to a cycle-run field test of young male national “Talent identification” and senior elite triathlon squads. J. Strength Cond. Res. 26, 2209–2219. 10.1519/JSC.0b013e31823a3c6b 21997447

[B12] Facer-Childs E. Brandstaetter R. (2015). The impact of circadian phenotype and time since awakening on diurnal performance in athletes. Curr. Biol. 25, 518–522. 10.1016/j.cub.2014.12.036 25639241

[B13] Giles L. V. Carlsten C. Koehle M. S. (2012). The effect of pre-exercise diesel exhaust exposure on cycling performance and cardio-respiratory variables. Inhal. Toxicol. 24, 783–789. 10.3109/08958378.2012.717649 23033992

[B14] Gronfier C. Wright K. P. Jr. Kronauer R. E. Jewett M. E. Czeisler C. A. (2004). Efficacy of a single sequence of intermittent bright light pulses for delaying circadian phase in humans. Am. J. Physiol. Endocrinol. Metab. 287, E174–E181. 10.1152/ajpendo.00385.2003 15039146 PMC2761596

[B15] Hébert M. Martin S. Lee C. Eastman C. (2002). The effects of prior light history on the suppression of melatonin by light in humans. J. Pineal Res. 33, 198–203. 10.1034/j.1600-079X.2002.01885.x 12390501 PMC3925650

[B16] Hoffmann G. Gufler V. Griesmacher A. Bartenbach C. Canazei M. Staggl S.. (2008a). Effects of variable lighting intensities and colour temperatures on sulphatoxymelatonin and subjective mood in an experimental office workplace. Appl. Ergon. 39, 719–728. 10.1016/j.apergo.2007.11.005 18164275

[B17] Hoffmann G. Leichtfried V. Griesmacher A. Bartenbach C. Canazei M. Staggl S. (2008b). Effects of light with reduced short wavelength components on parameters of circadian rhythm and performance in an experimental night shift model. Open Physiol. J. 1, 34–43. 10.2174/1874360900901010034

[B18] Kaida K. Takahashi M. Åkerstedt T. Nakata A. Otsuka Y. Haratani T.. (2006). Validation of the Karolinska sleepiness scale against performance and EEG variables. Clin. Neurophysiol. 117, 1574–1581. 10.1016/j.clinph.2006.03.011 16679057

[B19] Kantermann T. Forstner S. Halle M. Schlangen L. Roenneberg T. Schmidt-Trucksäss A. (2012). The stimulating effect of bright light on physical performance depends on internal time. PLoS ONE 7:e40655. 10.1371/journal.pone.0040655 22808224 PMC3394763

[B20] Knaier R. Meister S. Aeschbacher T. Gemperle D. Rossmeissl A. Cajochen C.. (2016). Dose–response relationship between light exposure and cycling performance. Scand. J. Med. Sci. Sports 26, 794–801. 10.1111/sms.12535 26271769

[B21] Lucas R. J. Peirson S. N. Berson D. M. Brown T. M. Cooper H. M. Czeisler C. A.. (2014). Measuring and using light in the melanopsin age. Trends Neurosci. 37, 1–9. 10.1016/j.tins.2013.10.004 24287308 PMC4699304

[B22] Midgley A. W. Bentley D. J. Luttikholt H. McNaughton L. R. Millet G. P. (2008). Challenging a dogma of exercise physiology: does an incremental exercise test for valid VO 2 max determination really need to last between 8 and 12 minutes? Sports Med. 38, 441–447. 10.2165/00007256-200838060-00001 18489192

[B23] O'Brien P. M. O'Connor P. J. (2000). Effect of bright light on cycling performance. Med. Sci. Sports Exerc. 32, 439–447. 10.1097/00005768-200002000-00027 10694129

[B24] Ohkuwa T. Itoh H. Yamamoto T. Yanagi H. Yamazaki Y. Akimaru T. (2001). Effect of varying light intensity on maximal power production and selected metabolic variables. Arch. Physiol. Biochem. 109, 430–434. 10.1076/apab.109.5.430.8034 11935384

[B25] Reilly T. Waterhouse J. (2009). Sports performance: is there evidence that the body clock plays a role? Eur. J. Appl. Physiol. 106, 321–332. 10.1007/s00421-009-1066-x 19418063

[B26] Revell V. L. Burgess H. J. Gazda C. J. Smith M. R. Fogg L. F. Eastman C. I. (2006). Advancing human circadian rhythms with afternoon melatonin and morning intermittent bright light. J. Clin. Endocrinol. Metab. 91, 54–59. 10.1210/jc.2005-1009 16263827 PMC3841985

[B27] Roenneberg T. Kuehnle T. Pramstaller P. Ricken J. Havel M. Guth A.. (2004). A marker for the end of adolescence. Curr. Biol. 14, R1038–R1039. 10.1016/j.cub.2004.11.039 15620633

[B28] Rüger M. Hilaire M. A. Brainard G. C. Khalsa S.-B. S. Kronauer R. E. Czeisler C. A.. (2013). Human phase response curve to a single 6.5 h pulse of short-wavelength light. J. Physiol. 591, 353–363. 10.1113/jphysiol.2012.239046 23090946 PMC3630790

[B29] Schmidt C. Collette F. Cajochen C. Peigneux P. (2007). A time to think: Circadian rhythms in human cognition. Cogn. Neuropsychol. 24, 755–789. 10.1080/02643290701754158 18066734

[B30] Smith M. R. Revell V. L. Charmane I. E. (2009). Phase advancing the human circadian clock with blue-enriched polychromatic light. Sleep Med. 10, 287–294. 10.1016/j.sleep.2008.05.005 18805055 PMC2723863

[B31] Steinacker J. M. Liu Y. Reissnecker S. (2002). Standards der Sportmedizin Ergometrie. Dtsch. Z. Für Sportmed. 53, 228–229.

[B32] Thompson A. Jones H. Marqueze E. Gregson W. Atkinson G. (2015). The effects of evening bright light exposure on subsequent morning exercise performance. Int. J. Sports Med. 36, 101–106. 10.1055/s-0034-1389970 25285469

[B33] Tucker R. Lambert M. I. Noakes T. D. (2006). An analysis of pacing strategies during men's world-record performances in track athletics. Int. J. Sports Physiol. Perform. 1, 233–245. 10.1123/ijspp.1.3.233 19116437

[B34] Vandewalle G. Schmidt C. Albouy G. Sterpenich V. Darsaud A. Rauchs G.. (2007). Brain responses to violet, blue, and green monochromatic light exposures in humans: prominent role of blue light and the brainstem. PLoS ONE 2:e1247. 10.1371/journal.pone.0001247 18043754 PMC2082413

[B35] Vickers A. J. Altman D. G. (2001). Analysing controlled trials with baseline and follow up measurements. BMJ 323, 1123–1124. 10.1136/bmj.323.7321.1123 11701584 PMC1121605

[B36] Wenger D. H. Bell G. J. (1986). The interactions of intensity, frequency and duration of exercise training in altering cardiorespiratory fitness. Sports Med. 3, 346–356. 10.2165/00007256-198603050-00004 3529283

[B37] Wirz-Justice A. Kräuchi K. Cajochen C. Danilenko K. V. Renz C. Weber J. M. (2004). Evening melatonin and bright light administration induce additive phase shifts in dim light melatonin onset. J. Pineal Res. 36, 192–194. 10.1111/j.1600-079X.2004.00117.x 15009510

[B38] Zeitzer J. M. Dijk D.-J. Kronauer R. E. Brown E. N. Czeisler C. A. (2000). Sensitivity of the human circadian pacemaker to nocturnal light: melatonin phase resetting and suppression. J. Physiol. 526, 695–702. 10.1111/j.1469-7793.2000.00695.x 10922269 PMC2270041

